# Apparatus for Histological Validation of *In Vivo* and *Ex Vivo* Magnetic Resonance Imaging of the Human Prostate

**DOI:** 10.3389/fonc.2017.00047

**Published:** 2017-03-24

**Authors:** Roger M. Bourne, Colleen Bailey, Edward William Johnston, Hayley Pye, Susan Heavey, Hayley Whitaker, Bernard Siow, Alex Freeman, Greg L. Shaw, Ashwin Sridhar, Thomy Mertzanidou, David J. Hawkes, Daniel C. Alexander, Shonit Punwani, Eleftheria Panagiotaki

**Affiliations:** ^1^Discipline of Medical Radiation Sciences, Faculty of Health Sciences, University of Sydney, Sydney, NSW, Australia; ^2^Centre for Medical Image Computing, University College London, London, UK; ^3^Centre for Medical Imaging, University College London, London, UK; ^4^Centre for Molecular Intervention, University College London, London, UK; ^5^Centre for Advanced Biomedical Imaging, University College London, London, UK; ^6^Department of Research Pathology, University College London, London, UK; ^7^Division of Surgery and Interventional Science, University College London, London, UK; ^8^Department of Urology, University College London Hospitals, London, UK

**Keywords:** prostate, prostate cancer, MRI validation, *in vivo* MRI, *ex vivo* MRI, histology, co-registration, fixation

## Abstract

This article describes apparatus to aid histological validation of magnetic resonance imaging studies of the human prostate. The apparatus includes a 3D-printed patient-specific mold that facilitates aligned *in vivo* and *ex vivo* imaging, *in situ* tissue fixation, and tissue sectioning with minimal organ deformation. The mold and a dedicated container include MRI-visible landmarks to enable consistent tissue positioning and minimize image registration complexity. The inclusion of high spatial resolution *ex vivo* imaging aids in registration of *in vivo* MRI and histopathology data.

## 1 The Need for Histological Validation of MRI

Imaging provides valuable non-invasive information for diagnosis and treatment of prostate cancer. The current state of the art is multi-parametric MRI (mp-MRI) (1), although even this lacks specificity and cannot provide reliable grading information. Advanced techniques (2, 3) show promise for probing cancer microstructure and may be more specific than conventional methods. However, rigorous validation is needed to assess the current and potential value of such techniques in prostate cancer management. The current gold standard for validation is histological assessment of whole mount serial sectioned radical prostatectomy specimens, but comparing information from such disparate images presents several challenges.

Below we give an overview of these challenges and the current methods for addressing some of them. Then, we describe a mold-based apparatus and imaging protocol that include *ex vivo* imaging and a number of innovations that improve alignment of imaging and histology planes and minimize in-plane rotational differences to improve the quality of the 2D registration processes. The apparatus also allows for the collection of supplementary *ex vivo* MR data for both the fresh unfixed and the fixed prostate specimen.

## 2 Problems in Histological Validation of MRI Methods

There are a number of major technical problems complicating any attempt to directly match and correlate MRI and histology data (4). These difficulties include

- **Unmatched tissue planes**. In practice there is usually no direct coordination of presurgical imaging methods and surgical specimen processing. This results in MRI slice planes usually having different plane orientation and plane spacing from the histology images with no clear correspondence between the two so that, at best, only a qualitative correlation of MRI and pathology data is feasible.
- **Unmatched spatial resolution**. A volume element (“voxel”) in prostate MRI (the source of each semi-discrete item of measurement data) has a typical size that may vary from 0.2 mm × 0.2 mm in-plane by 1 mm slice thickness to 2 mm × 2 mm in-plane by 5 mm slice thickness. By contrast, a typical histological image used for pathology assessment is based on a 3–5 µm thick tissue section and has an in-plane resolution <1 µm. Even under highly idealized conditions, where the histology and MRI slices are “co-planar,” the MRI signal originates from a much larger tissue volume than is represented in the histology slice. Thus, there may be tissue structure heterogeneity within the MRI slice that is not reflected in the histological data.
- **Tissue deformations**. The prostate is deformed *in vivo* by voluntary and involuntary body movements, bowel contents, and by the imaging system itself if an endorectal coil is employed for signal detection. Upon resection, the prostate is detached from its supporting tissues and associated vasculature, resulting in shape changes due to removal of mechanical tension and compression and hemodynamic pressure (5). Dehydration and embedding of the tissue during processing for histology results in tissue shrinkage and thin sectioning may cause further deformations.
- **Unmatched image features**. MRI and histological staining produce image contrast according to very different tissue properties. The features present in MR images may not be easily identified in histology images and *vice versa*, leading to a difficulty in assessing the accuracy of any co-registration process.

## 3 Survey of Methods

The validation problems mentioned earlier have been addressed with varying degrees of completeness by various methods, some of which have been reviewed in Ref. (4, 6). Here, we provide a brief overview of the approaches and problems addressed by the method we describe.

A partial 3D histology “map” can be reconstructed from a stack of histology slices and registered with MR images by a 3D process (7, 8); however, the accuracy of this approach is severely limited by the low out of plane resolution of the MRI data. The problem of missing inter-plane histology data can be addressed by manual (9, 10) or automatic (11) selection of the most closely aligned histology and image planes. Ideally, this reduces the problematic 3D registration to a more tractable 2D process. As the accuracy of this approach is highly dependent on co-alignment of imaging planes with histology sections, a number of methods have been described based on production of a patient-specific 3D-printed mold, the shape of which is defined by the *in vivo* imaging data. The aim of the mold is to hold the prostate in the same conformation in which it was imaged while guides in the mold align the tissue cutting planes with the image slice positions (12–15). The precision of these mold-based methods is dependent on the degree to which the mold matches the shape of the excised prostate specimen and the amount of any mispositioning or rotation of the prostate in the mold. Rotation errors are more likely around the axes in which the specimen has greatest rotational symmetry. Inclusion of a urethral catheter in the specimen and mold design can decrease rotation errors about the left–right axes of the prostate (6).

High spatial resolution *ex vivo* imaging of the prostate specimen has been used as an intermediate “stepping-stone” to improve the accuracy of co-registration of *in vivo* MRI and histology data, both with (6) and without (16) a patient-specific mold. However, the mold design can be improved to better facilitate comparison between MR and histological images.

## 4 Outline of Imaging and Specimen Handling

The following points outline the method of imaging and construction of a patient-specific 3D-printed mold to optimize physical location of the prostate in *in vivo* MR images, *ex vivo* MR images, and histology images. The aim is to reduce the inherent 3-dimensional co-registration problem to a more tractable and precise 2-dimensional process.

### 4.1 *In Vivo* Imaging

This study was carried out in accordance with the recommendations of the UK Research Governance Framework version 2, UK Research Ethics Committee with written informed consent from all subjects and approved by the NRES Committee London-Surrey Borders (REC 15/LO/0692). All subjects gave written informed consent in accordance with the Declaration of Helsinki.

The geometry for the *in vivo* imaging needs to be compatible with the local clinical histopathology processing and reporting protocol. In our case, the pathology department uses 5 mm thick sections cut approximately transaxial to the prostatic urethra and perpendicular to the posterior face of the prostate. We perform T2-weighted imaging with 2.5 mm slice thickness (with no gap or 2.5 mm gap) in the “true axial” scanner XY plane. The central slice is centered approximately mid-organ and is defined as the reference for all subsequent imaging and processing and is defined by MR visible landmarks in the patient-specific mold.

For the data illustrating the methods in this paper mp-MRI was performed on a 3-T scanner (Achieva, Philips, Best, the Netherlands), using pelvic phased array coils. The 0.2 mg/kg (up to 20 mg) of a spasmolytic agent (Buscopan; Boehringer Ingelheim, Germany) was administered intravenously prior to imaging to reduce bowel peristalsis. mp-MRI comprises axial and coronal T2 turbo spin echo (TSE) imaging, supplemented with diffusion-weighted imaging at *b*-values 0, 150, 500, and 1,000 s/mm^2^. A dynamic contrast enhanced (DCE) acquisition was subsequently performed using spoiled gradient echo with fat saturation and a 12-s time resolution. Intravenous contrast agent (0.2 mL/kg; Prohance, Bracco, Milan, Italy) was injected at the beginning of the 6th acquisition at 3 mL/s followed by 20 mL of saline.

### 4.2 Contouring of *In Vivo* Images for Patient-Specific Mold Specification

MR datasets were analyzed using Osirix Version 7.0 (Bernex, Switzerland). A board certified Radiologist (Edward William Johnston) manually contoured the entire prostate (from base to apex) using the closed polygon tool on high-resolution axial T2-weighted images. Contoured landmarks comprise the hypointense prostate capsule and the anterior fibromuscular stroma where the capsule is absent anteriorly. The periprostatic fat, neurovascular bundles, seminal vesicles, distal urethral sphincter, and bladder were not included inside the contoured volume but provided useful information as to the extent of the prostate. The positions of contours were checked in all planes and adjusted accordingly until their appearance was satisfactory. Where available, at least 3 slices of coronal and/or sagittal images were contoured at the center of the gland to corroborate axial contours, but the edge of the prostate was not contoured as it is poorly delineated in these planes. To estimate urethral catheter position, the urethra was demarcated where visible using axial T2-weighted images and interpolating based on normal anatomy and adjacent slice position where invisible.

### 4.3 Prostatectomy and Specimen Preparation

The radical prostatectomy specimen was collected immediately upon resection to minimize ischemia time and taken to the pathology department without formalin fixation. The fresh specimen was inked and dried, and the seminal vesicles and any metal clips that would cause magnetic susceptibility artifacts were removed. A 4.2-mm diameter silicon rubber catheter was inserted through the urethra, the prostate placed into the mold, and the mold halves fastened with four plastic cable ties. The specimen inside the closed mold was then inserted into the canister, which had been pre-filled with saline. Gentle agitation was used to eliminate air bubbles, and then the sealing piston was inserted fully to ensure alignment of the mold reference plane with the external reference landmarks. Excess fluid was ejected through the vent, which was then capped.

### 4.4 *Ex Vivo* Prostate Imaging

Fresh *ex vivo* scanning was performed on both a 3-T clinical MRI scanner (Philips Achieva, Best, the Netherlands) and a 9.4-T 20-cm horizontal bore MRI (Varian Inc., Palo Alto, CA, USA). Fixed imaging was conducted only at 9.4 T. The reference plane landmarks on the exterior of the mold canister (see section 6 below) were used to position the reference slice of the sample near the isocenter of the magnet.

For 3-T MRI, the sample was positioned at the center of an 8-channel knee coil (Philips, Best). T2-weighted TSE images (TE = 100 ms, echo train length = 16, TR = 5.2 s, field of view 18 cm × 18 cm) were acquired with 0.4 mm × 0.4 mm resolution in-plane, 2 mm slice thickness, and 0.5 mm slice gap.

**Figure 1:**
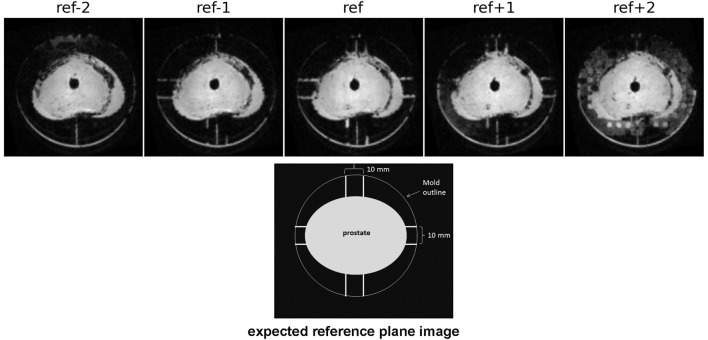
**Images acquired at 1 mm spacing demonstrate the use of the reference plane landmark channels (some of which are obscured by air bubbles here) to locate the reference slice**. When correctly positioned and aligned the reference image will show the water filled channels on the A/P and L/R sides of the prostate.

Imaging at 9.4 T was performed using 400 mT/m gradients and a 72 mm internal diameter quadrature coil (RAPID Biomedical, Rimpar, Germany). A multi-slice gradient echo sequence (TE = 5 ms, TR = 75 ms, field of view 8 cm × 8 cm) was acquired giving 0.625 mm × 0.625 mm in-plane resolution, 1 mm slice thickness, and no slice gap. These images were used to locate the reference slice using the reference plane landmarks (see section 5.1 below), as shown in Figure 1. The same procedure for reference slice location was repeated following specimen fixation.

## 5 Mold Design and 3D Printing

### 5.1 Mold Template

**Figure 2:**
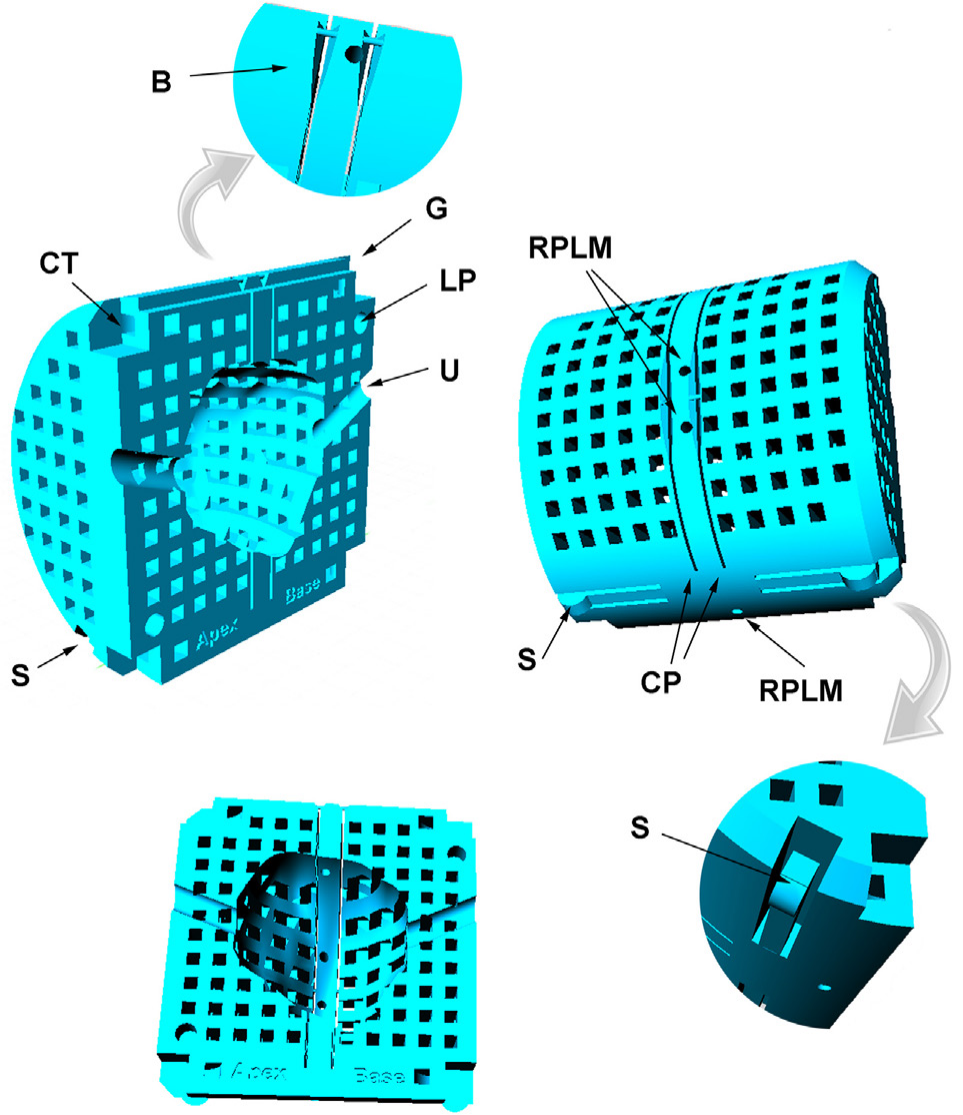
**Mold design features**. See text for label descriptions.

The mold template is 62 mm diameter, designed to fit inside a 63.5 mm internal diameter canister that contains the prostate immersed in saline during *ex vivo* imaging. The mold design is based on the abilities and constraints of nylon powder printing (selective laser sintering using EOSINT P100 with PA2200 powder; 40–50 µm grain size). Note that the mold design described here could not be produced on a typical low-cost plastic filament printer due to the lower dimensional precision and the need for these devices to print support material that would be extremely difficult to remove from the design we describe. A more basic mold design suitable for a filament printer is described in Ref. (6). The mold features are illustrated in Figure 2 and include

**Figure 3:**
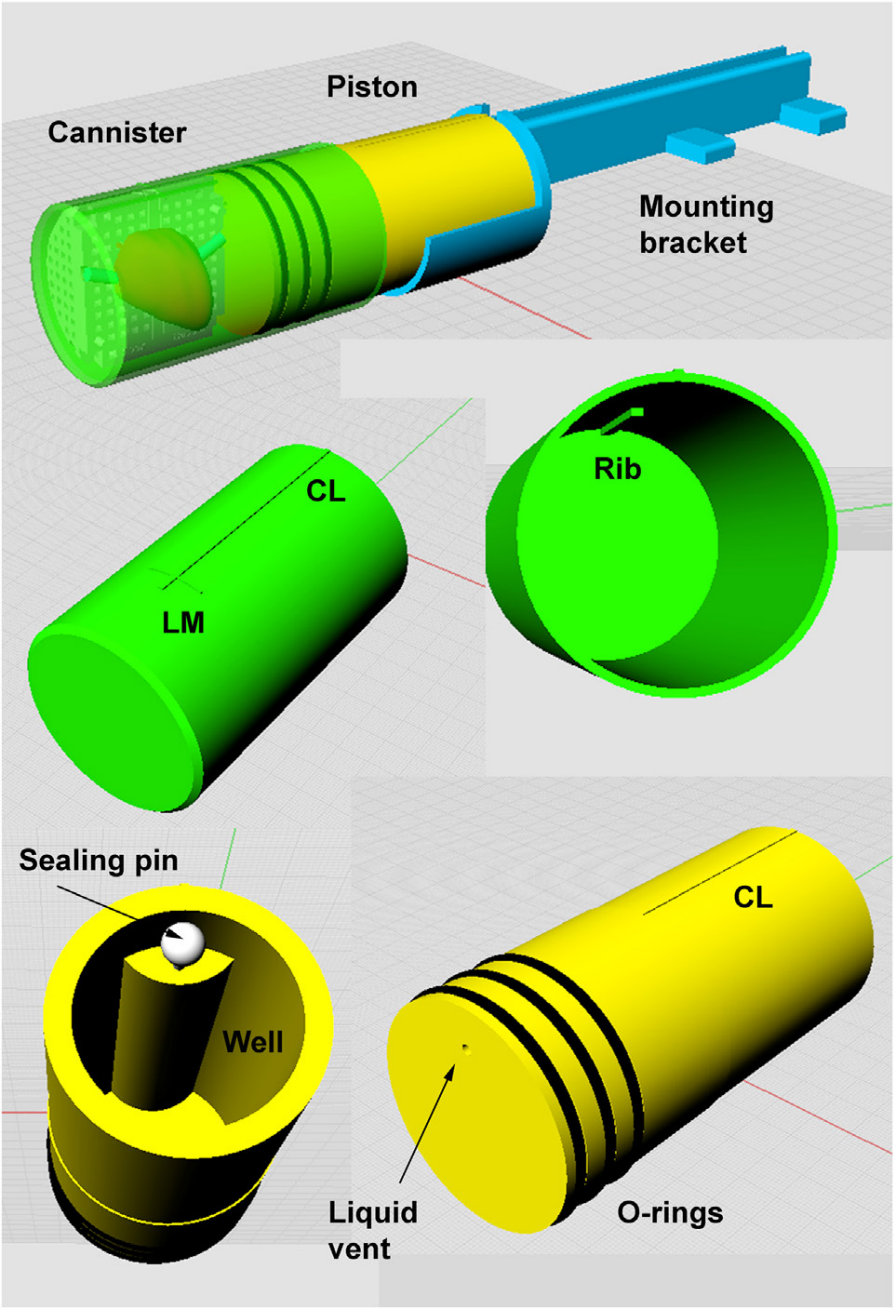
**Apparatus for *ex vivo* imaging of the prostate immersed in saline**. See text for design features description.

- A chamfer at one end of the mold indicates the apex end of the prostate, so that orientation of the prostate during *ex vivo* imaging is as for *in vivo* imaging with the patient lying in feet-first supine position.
- **LP**. Two 4-mm diameter locator pins ensure alignment of the mold halves.
- **CP**. Two 0.4-mm wide slots, spaced 5.0 mm apart, define the cutting planes for organ sectioning.
- **B**. 0.5-mm diameter braces stabilize the mold shape during *ex vivo* imaging. These are removed immediately prior to organ sectioning.
- **RPLM**. Reference plane land marks (1.5-mm diameter axial channels) are filled with liquid when the prostate and mold are inserted in the imaging canister and provide MR visible landmarks that enable precise matching of *in vivo* and *ex vivo* imaging planes. Relative to the cutting planes the landmarks are offset toward the apex of the prostate to bring the center of the MRI planes into closer alignment with the histology sections that are routinely cut from the apex face of the tissue blocks.
- **U**. A 4.2-mm diameter saline-filled silicon rubber urethral catheter is inserted in the prostate with curvature and orientation defined during contouring of the *in vivo* images. The catheter assists in constraining any rotations of the prostate in the sagittal plane.
- **S**. Flexible springs printed into the mold press against the wall of the canister and minimize any movement or vibration of the mold in the canister during imaging.
- **G**. A groove in the top of the mold matches the internal rib in the canister (Figure 3) and precludes any rotation of the mold in the axial plane.
- **CT**. The corners of the mold halves are fastened with four plastic cable ties.

## 6 Mold Container for *Ex Vivo* Imaging

For *ex vivo* imaging (both before and after formalin fixation), the specimen in the mold is constrained inside a plastic canister. A liquid-tight seal is made with a piston that also serves as the mounting base for attachment of the sample to the scanner’s small animal bed. Details are described in Figure 3 and below:

- **Canister**. The canister is 68 mm outside diameter, 63.5 mm internal, to fit with 2 mm clearance inside the 72 mm internal diameter RF coil used for 9.4-T *ex vivo* imaging. The canister is printed in rigid photopolymer (Objet, Stratasys), which is non-porous. Landmarks on the external surface of the canister define the position of the reference slice and top midline of the mold. An internal rib fits the groove in the top of the mold and prevents any rotation of the mold inside the canister, thus ensuring minimal axial plane rotational differences between *in vivo* and *ex vivo* images (see Figure 2).
- **Piston**. The canister is sealed liquid tight with a piston (also printed in rigid photopolymer) fitted with three O-rings lubricated with light grease. To immerse the prostate in liquid and minimize inclusion of air bubbles the canister is placed vertically and filled with 200 mL of saline. The prostate in the mold is inserted, pushed to the bottom of the canister, and agitated to expel air bubbles. The piston is then inserted and excess liquid is expelled into the central well via the vent which is then sealed with a plastic pin. The center line landmark (CL) on the piston is aligned with the center line mark on the canister. The fully inserted piston fixes the mold at the base of the canister, so that the mold reference plane is aligned with the external landmarks. The mold and prostate are thus fixed in a defined position relative to the external landmarks on the canister and piston.
- **Mounting bracket**. The base of the piston is attached to a mounting bracket that is designed specifically for the small animal bed of the MRI scanner. The bracket is printed on the SLS printer that provides a rigid product (slightly porous so unsuitable for canister and piston).

## 7 Formalin Fixation of Prostate in Mold

The porous mold design enables fixation of the prostate *in situ*. The mold/prostate is removed from the canister and immersed in 1-L fixative solution in a narrow vessel placed on a magnetic stirrer overnight. The low density of the nylon mold provides sufficient buoyancy for the prostate to float just below the liquid surface. Use of the stirrer enhances penetration of the fixative through the mold structure and into the prostate. Post fixation, the immersion solution is replaced with 1-L saline for 8 h to dilute the formalin which, at full strength, severely reduces the sample T2 leading to poor signal quality.

Formalin fixation leads to tissue shrinkage (17) and shape changes (18). Fixation of the prostate in the mold constrains any shape changes.

## 8 Apparatus for Sectioning Prostate in Mold

**Figure 4:**
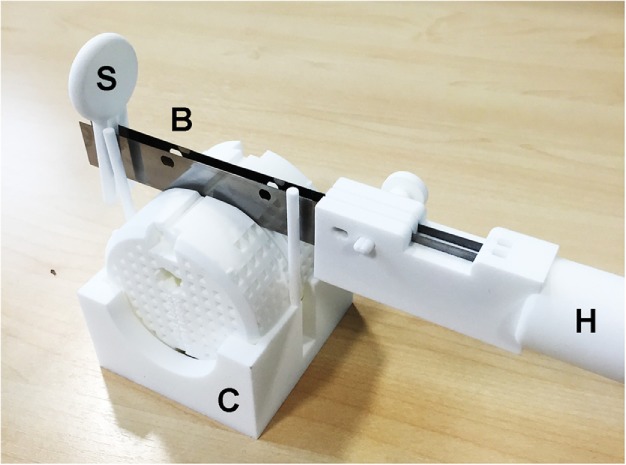
**Prostate slicing in mold**. The mold is stabilized in a cradle (C). The handle (H) and separate spacer (S) maintain two skin graft blades (B) at the same 5 mm spacing as the mold cutting planes.

Upon completion of fixation and *ex vivo* imaging, the prostate is sectioned for histological processing (Figure 4). The mold defines two cutting planes spaced 5 mm apart on either side of the imaging reference plane. Prior to cutting, the mold stabilizer braces (Figure 2) are removed with a scalpel blade, and the mold placed in a dedicated cradle (C). The two cuts on either side of the reference slice are made simultaneously using a pair of skin graft blades (R55170, Rocket Medical, Washington, UK) mounted in an SLS-printed handle (H). The handle and a detachable spacer (S) maintain the blades at 5-mm spacing that aids insertion of the blades into the slots in the top of the mold.

After cutting of the reference plane section, the mold is opened and the prostate removed. The base and apex volumes are then progressively sectioned at 5-mm thickness using a flat plate with 5-mm high rails to guide the blade.

Whole mount tissue sections are processed according to standard laboratory protocol. These sections are cut from the prostate apex face of each block. Note that the reference plane landmarks in the mold are offset toward the apex to account for the histology slice position typically being closer to the apex side of each tissue section than to the base side.

## 9 Image Registration

There is a very wide range of 2D image registration methods that could be applied for alignment of the MRI and histology images. As the focus of this paper is on the apparatus used for prostate imaging and sectioning, we present here only an outline of the methods used to produce the example data presented in the figures.

Hematoxylin and eosin stained whole mount sections were digitally scanned with a 20× objective (Hamamatsu NanoZoomer). Histological images were downsampled to 0.25× and converted to grayscale for registration. The high-resolution *ex vivo* T2 MRI slice for registration was selected based on the mold landmarks. Registration to the corresponding histological slice used an intensity-based 2D rigid registration based on a block-matching strategy (19, 20) with the correlation coefficient as similarity measure. The *ex vivo* MRI was registered to the *in vivo* MRI using 2D affine registration, restricting the block matching in the *in vivo* image to the prostate region using the contour from section 4.2.

## 10 Sample Results

**Figure 5:**
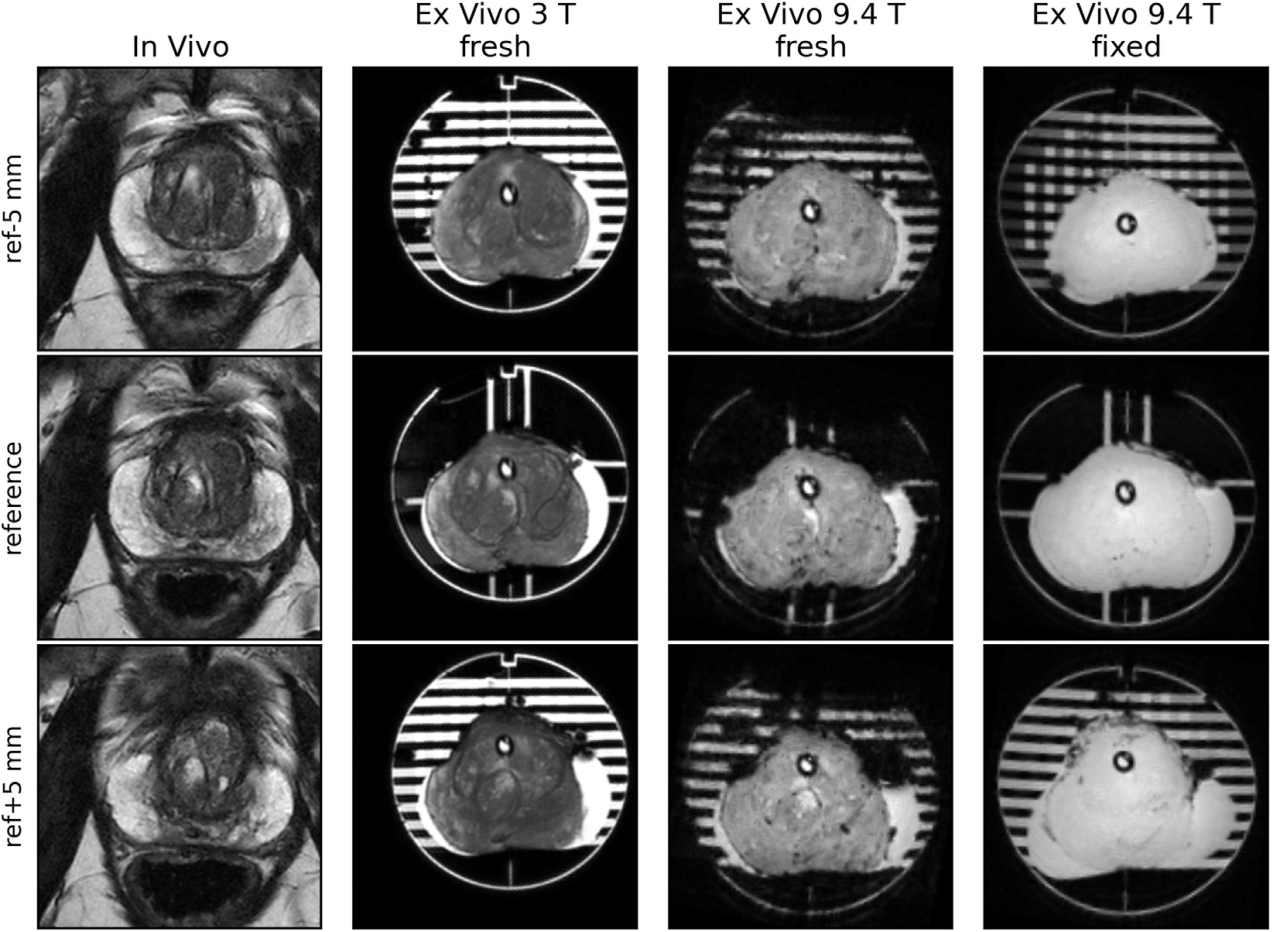
**Images from the reference slice and slices ±5 mm from the reference**. Contours from the *in vivo* scan’s reference slice were aligned with the reference plane of the mold. The 8 channels in the reference plane were used to locate the reference slice for fresh scans at 3 and 9.4 T, as well as after fixation.

An example case is shown in Figure 5.

**Figure 6:**
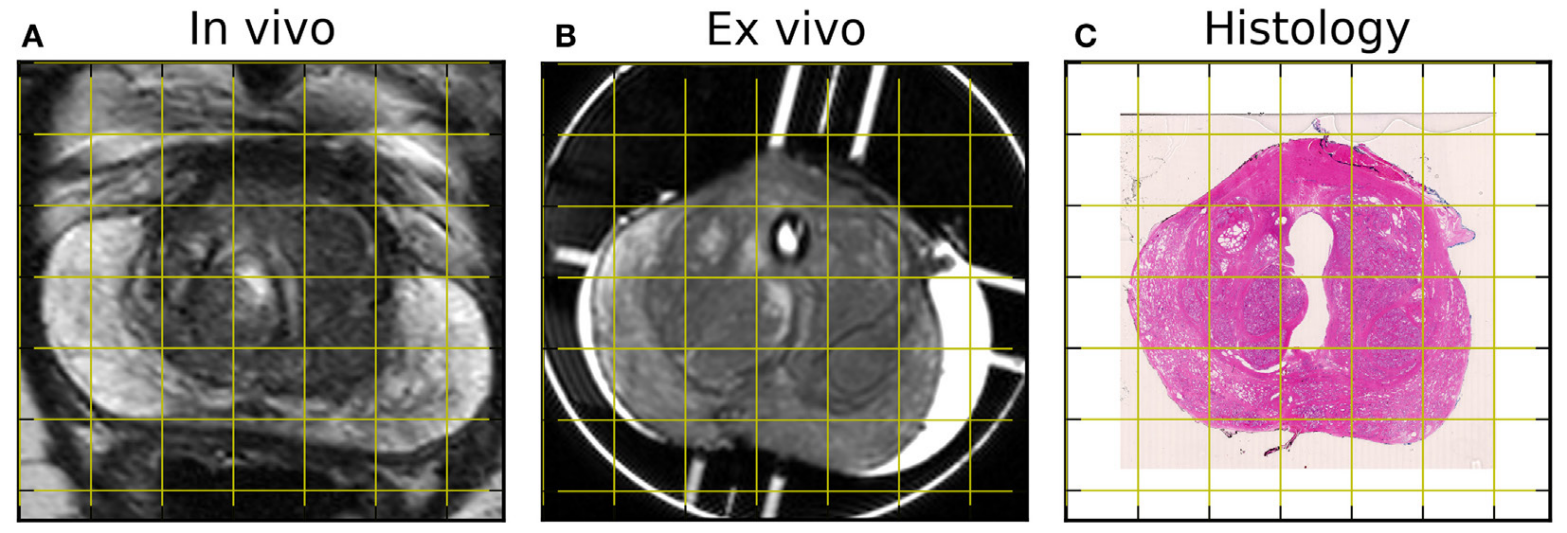
**Sample *in vivo* (A) and *ex vivo* (B) MR images after rigid registration to hematoxylin and eosin-stained histology (C)**. Note the collapse of the peripheral zone between *in vivo* and *ex vivo* scans, as well as the distortion around the urethra in the histological section.

Registered MRI and histology slices are shown in Figure 6. Slicing artifacts, particularly near the urethra, are visible and may be corrected by additional non-rigid registration techniques. Nevertheless, the spatial correspondence between the *ex vivo* and histology images can be seen, including in the peripheral zone shape and in the outline of glandular regions within the transition zone. The peripheral zone partially collapses between *in vivo* and *ex vivo* imaging, but internal structures within the transition zone show correspondence.

## 11 Limitations and Future Directions

The apparatus detailed here addresses many of the issues described in section 2 and extends previous methods by improving the alignment of *in vivo* and *ex vivo* imaging planes with each other and with histopathology sections. Nevertheless, a number of problems remain

- **Prostate contouring from *in vivo* images**. Contour delineation on the *in vivo* images aims to trace the expected surgical margins. These are normally close to the prostatic capsule but may vary case by case and the capsule is absent around the prostatic apex. Depending on the T2 image quality and characteristics of the individual prostate, it is sometimes difficult to discriminate the capsule from adjacent intra and extra-prostatic structures. One strategy to accommodate these uncertainties is to print multiple versions of the mold with +10 and −10% adjustments to the axial plane scale and to fit the resected specimen to the mold that provides the best fit.
- ***In vivo*–*ex vivo* volume changes**. In our experience, there may be significant prostate volume reductions upon resection (see Figure 5) due to loss of luminal fluid. This may lead to a loose and unconstrained fit of the prostate in the mold and consequent misalignment of imaging planes and tissue sectioning planes. Inclusion of the urethral catheter reduces the likelihood of such misalignment, although introducing a small distortion of the tissue around the catheter. As the majority of tumors are not proximal to the urethra, the latter is a relatively minor issue.
- **Volume changes due to fixation**. The mold constrains prostate movement during fixation, but does not account for volume shrinkage. It is possible that the orientation of the prostate within the mold could change due to the less close fit after shrinkage. This issue could potentially be addressed by transferring the fixed prostate to a second mold with reduced prostate volume; however, we have not implemented this step that assumes a predictable and uniform shrinkage.
- **Inconsistent MRI and histology section thickness**. The apparatus described improves the reliability of imaging plane alignment with histology planes but does not specifically address the problem of sparse histology data in the direction orthogonal to the sectioning planes. This is primarily a tissue processing issue and if resources permit can be addressed by more comprehensive thin sectioning and processing of the tissue blocks.

## Author Contributions

Apparatus design: RB. Specimen preparation and handling: GS, AS, CB, SH, HP, and AF. Imaging: EJ, CB, and BS. Image registration: TM and CB. Project management: DA, DH, SP, and EP. Manuscript preparation: all.

## Conflict of Interest Statement

The authors declare that the research was conducted in the absence of any commercial or financial relationships that could be construed as a potential conflict of interest.
